# 1,2,5-Oxadiazolo[3,4-*b*]glycyrrhetinic acid

**DOI:** 10.1107/S1600536809020996

**Published:** 2009-06-10

**Authors:** Jun Hu, Libing Yu, Ruji Wang, Yong Ju

**Affiliations:** a Key Laboratory of Bioorganic Phosphorus Chemistry & Chemical Biology, Ministry of Education, Department of Chemistry, Tsinghua University, Beijing 100084, People’s Republic of China

## Abstract

The title compound [systematic name: 11-oxo-2,3-(oxy­dinitrilo)olean-12-en-29-oic acid], C_30_H_42_N_2_O_4_, contains a linear array of five six-membered rings and a five-membered heterocyclic ring. The *C* ring, containing an α,β-unsaturated ketone, has a slightly distorted half-chair conformation, as does the *A* ring, with N—C—C angles 125.3 (5), 111.2 (4), 124.9 (5) and 109.2 (5)°, while the other three six-membered rings adopt chair conformations. The enanti­omer has been assigned by reference to unchanging chiral centres in the synthetic procedure. An intramolecular C—H⋯O interaction is present. In the crystal structure, inter­molecular O—H⋯O hydrogen bonds link the mol­ecules.

## Related literature

Glycyrrhetinic acid is the aglycone of glycyrrhizin, a triterpenoid saponin found in the roots of liquorice, see: Yoshida *et al.* (2001 ▶). For the pharmacological activities of glycyrrhetinic acid and its derivatives, see: Finney & Tarknoy (1960 ▶); Yu *et al.* (2006 ▶); Su *et al.* (2004 ▶).
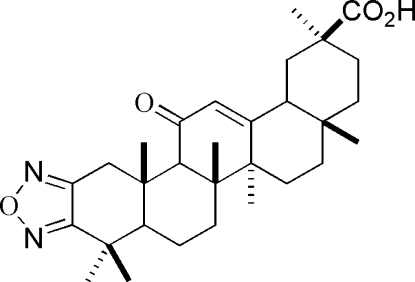


## Experimental

### 

#### Crystal data

C_30_H_42_N_2_O_4_
*M* *_r_* = 494.66
Orthorhombic, 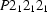

*a* = 8.5489 (11) Å
*b* = 10.9019 (18) Å
*c* = 28.898 (3) Å
*V* = 2693.2 (6) Å^3^
*Z* = 4
Mo *K*α radiation
μ = 0.08 mm^−1^
*T* = 295 K
0.5 × 0.4 × 0.3 mm

#### Data collection

Bruker P4 diffractometer
Absorption correction: none
3717 measured reflections
2855 independent reflections
2178 reflections with *I* > 2σ(*I*)
*R*_int_ = 0.031
3 standard reflections every 97 reflections intensity decay: none

#### Refinement

*R*[*F* ^2^ > 2σ(*F* ^2^)] = 0.049
*wR*(*F* ^2^) = 0.109
*S* = 1.02
2855 reflections
326 parameters
H-atom parameters constrained
Δρ_max_ = 0.16 e Å^−3^
Δρ_min_ = −0.18 e Å^−3^

### 

Data collection: *XSCANS* (Bruker, 1997 ▶); cell refinement: *XSCANS*; data reduction: *XSCANS*; program(s) used to solve structure: *SHELXTL* (Sheldrick, 2008 ▶); program(s) used to refine structure: *SHELXTL*; molecular graphics: *SHELXTL*; software used to prepare material for publication: *SHELXTL*.

## Supplementary Material

Crystal structure: contains datablocks I, global. DOI: 10.1107/S1600536809020996/at2805sup1.cif

Structure factors: contains datablocks I. DOI: 10.1107/S1600536809020996/at2805Isup2.hkl

Additional supplementary materials:  crystallographic information; 3D view; checkCIF report

## Figures and Tables

**Table 1:** Hydrogen-bond geometry (Å, °)

*D*—H⋯*A*	*D*—H	H⋯*A*	*D*⋯*A*	*D*—H⋯*A*
O4—H4*A*⋯O2^i^	0.82	2.04	2.787 (4)	151
C1—H1*A*⋯O2	0.97	2.36	2.969 (5)	120
C25—H25*B*⋯O2	0.96	2.39	3.021 (5)	123

Symmetry code: (i) 
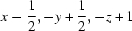
.

**Fig. 1.**
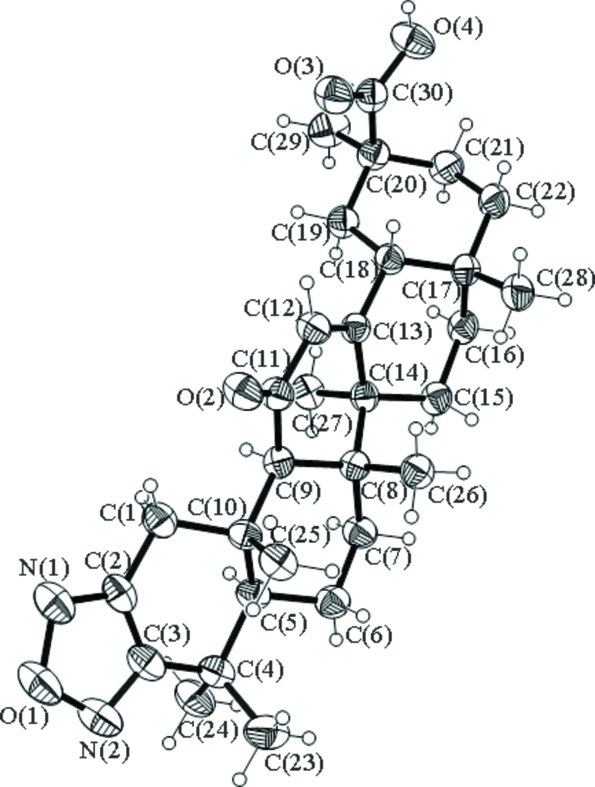
ORTEP drawing of title compound with 35% probability ellipsoids, showing the atomic numbering scheme.

**Fig. 2.**
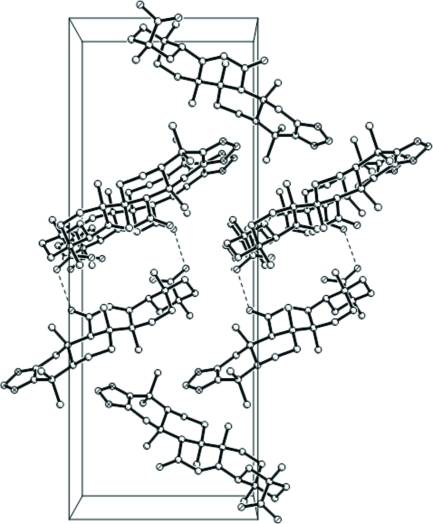
A packing view along the a direction.

## supplementary crystallographic information

### Comment

Glycyrrhetinic acid is the aglycone of glycyrrhizin, a triterpenoid saponin found in the roots of licorice(glycyrrhiza glabra)(Yoshida *et al.*, 2001). It is well known as a potent sweet saponin and a number of triterpenoids. Glycyrrhetinic acid and its derivatives have several pharmacological activities, such as anti-inflammatory(Finney *et al.*, 1960), anti-viral(Yu *et al.*, 2006) and anti-tumor(Su *et al.* 2004). Recently, our group has designed and synthesized a novel series of glycyrrhetinic acid derivatives by chemical modification, and [2,3-*c*][1,2,5]oxadiazol-glycyrrhetinic acid showed primarily considerable bioactivity of inhibiting growth of HepG-2 tumour cell.

The title compound was obtained as colourless plate in the orthorhombic space group P2(1)2(1)2(1). A view of the molecular structure with the numbering scheme is shown in Fig. 1 and selected dimensions are given in Table 1. The molecule is composed of five fused six-membered rings and a five-membered heterocyclic ring, *viz*. A(C1–C5/C10), B(C5–C10), C(C8/C9/C11–C14), D(C13–C18), E(C17–C22) and F(C2—N2—O2—N1—C3). Ring B, D and E adopt chair conformations, while ring A and C adopt a slightly distorted half-chair conformation as a result of the double bonds being in or out of rings. The lengths of single bonds N1–C2 and N2–C3 are nearly equal, though there is a gem-dimethyl on C4.

### Experimental

2,3-dihydroxyimino-glycyrrhetinic acid (500 mg, 0.98 mmol), 18-Crown-6 (35 mg, 0.13 mmol) and t-BuOK (220 mg, 1.96 mmol) were dissolved in 15 mL THF and 15 mL DCM. The mixture was stirred for 10 min at 50°C. The product was treated with hydrochloric acid, then washed with H_2_O and extracted with EtOAc. The combined organic phase was washed with brine, dried and evaporated. Purification by flash chromatography(DCM:Methanol=20:1) gave title compound(439 mg, 91%). Crystals suitable for X-ray structure analysis were obtained *via* slow evaporation of a solution(chloroform: acetone = 1:7) at room temperature.

### Refinement

All non-hydrogen atoms were subjected to anisotropic refinement. All hydrogen atoms were generated geometrically with O—H bond distance of 0.82 Å and C—H bonds of 0.93–0.98 Å according to criteria described in the *SHELXTL* manual (Sheldrick, 2008). They were included in the refinement with *U*_iso_(H) = 1.2*U*_eq_ or 1.5*U*_eq_ (for methyl C). Final difference Fourier maps showed the highestand lowest electron densities of -0.182 and 0.157 *e* Å^-3^, respectively. Friedel pairs were averaged in the final refinement.

### Crystal data

**Table:**

C_30_H_42_N_2_O_4_	*F*(000) = 1072
*M**_r_* = 494.66	*D*_x_ = 1.220 Mg m^−^^3^
Orthorhombic, *P*2_1_2_1_2_1_	Mo *K*α radiation, λ = 0.71073 Å
Hall symbol: P 2ac 2ab	Cell parameters from 52 reflections
*a* = 8.5489 (11) Å	θ = 3.7–19.0°
*b* = 10.9019 (18) Å	µ = 0.08 mm^−^^1^
*c* = 28.898 (3) Å	*T* = 295 K
*V* = 2693.2 (6) Å^3^	Prism, colorless
*Z* = 4	0.5 × 0.4 × 0.3 mm

### Data collection

**Table:**

Bruker P4 diffractometer	*R*_int_ = 0.031
Radiation source: fine-focus sealed tube	θ_max_ = 25.5°, θ_min_ = 2.0°
graphite	*h* = −1→10
ω scans	*k* = −13→1
3717 measured reflections	*l* = −1→35
2855 independent reflections	3 standard reflections every 97 reflections
2178 reflections with *I* > 2σ(*I*)	intensity decay: none

### Refinement

**Table:**

Refinement on *F*^2^	Primary atom site location: structure-invariant direct methods
Least-squares matrix: full	Secondary atom site location: difference Fourier map
*R*[*F*^2^ > 2σ(*F*^2^)] = 0.049	Hydrogen site location: inferred from neighbouring sites
*wR*(*F*^2^) = 0.109	H-atom parameters constrained
*S* = 1.02	*w* = 1/[σ^2^(*F*_o_^2^) + (0.001*P*)^2^ + 1.3*P*] where *P* = (*F*_o_^2^ + 2*F*_c_^2^)/3
2855 reflections	(Δ/σ)_max_ < 0.001
326 parameters	Δρ_max_ = 0.16 e Å^−^^3^
0 restraints	Δρ_min_ = −0.18 e Å^−^^3^

### Special details

**Table:**

Experimental. absolute configuration has not been established by anomalous dispersion effects in diffraction measurements on the crystal. The enantiomer has been assigned by reference to unchanging chiral centres in the synthetic procedure.
Geometry. All e.s.d.'s (except the e.s.d. in the dihedral angle between two l.s. planes) are estimated using the full covariance matrix. The cell e.s.d.'s are taken into account individually in the estimation of e.s.d.'s in distances, angles and torsion angles; correlations between e.s.d.'s in cell parameters are only used when they are defined by crystal symmetry. An approximate (isotropic) treatment of cell e.s.d.'s is used for estimating e.s.d.'s involving l.s. planes.
Refinement. Refinement of *F*^2^ against ALL reflections. The weighted *R*-factor *wR* and goodness of fit *S* are based on *F*^2^, conventional *R*-factors *R* are based on *F*, with *F* set to zero for negative *F*^2^. The threshold expression of *F*^2^ > σ(*F*^2^) is used only for calculating *R*-factors(gt) *etc*. and is not relevant to the choice of reflections for refinement. *R*-factors based on *F*^2^ are statistically about twice as large as those based on *F*, and *R*- factors based on ALL data will be even larger.

### Fractional atomic coordinates and isotropic or equivalent isotropic displacement parameters (Å^2^)

**Table:**

	*x*	*y*	*z*	*U*_iso_*/*U*_eq_	
O1	0.6409 (6)	−0.3844 (3)	0.25922 (12)	0.0902 (11)	
O2	0.4151 (4)	−0.0311 (2)	0.41200 (9)	0.0636 (9)	
O3	0.0227 (4)	0.4128 (3)	0.47645 (11)	0.0733 (9)	
O4	0.0196 (5)	0.6051 (3)	0.50103 (11)	0.0802 (10)	
H4A	−0.0380	0.5758	0.5208	0.096*	
N1	0.5282 (6)	−0.3188 (3)	0.28470 (14)	0.0767 (12)	
N2	0.7751 (6)	−0.3138 (4)	0.25298 (14)	0.0826 (14)	
C1	0.5253 (6)	−0.1096 (4)	0.31919 (14)	0.0592 (12)	
H1A	0.4552	−0.1415	0.3427	0.071*	
H1B	0.4653	−0.0574	0.2986	0.071*	
C2	0.5954 (6)	−0.2133 (4)	0.29242 (14)	0.0587 (12)	
C3	0.7457 (6)	−0.2096 (4)	0.27309 (14)	0.0621 (13)	
C4	0.8579 (6)	−0.1039 (4)	0.27610 (14)	0.0586 (12)	
C5	0.7758 (5)	0.0033 (4)	0.30352 (13)	0.0535 (11)	
H5A	0.7143	0.0485	0.2805	0.064*	
C6	0.8964 (5)	0.0951 (4)	0.32221 (14)	0.0585 (11)	
H6A	0.9502	0.0594	0.3485	0.070*	
H6B	0.9734	0.1123	0.2985	0.070*	
C7	0.8171 (5)	0.2144 (4)	0.33693 (14)	0.0573 (11)	
H7A	0.7717	0.2530	0.3098	0.069*	
H7B	0.8959	0.2696	0.3492	0.069*	
C8	0.6882 (5)	0.1969 (4)	0.37362 (13)	0.0462 (10)	
C9	0.5807 (5)	0.0872 (3)	0.36035 (13)	0.0466 (10)	
H9A	0.5194	0.1173	0.3340	0.056*	
C10	0.6578 (5)	−0.0339 (3)	0.34242 (13)	0.0483 (10)	
C11	0.4616 (5)	0.0705 (4)	0.39885 (14)	0.0526 (11)	
C12	0.3977 (6)	0.1813 (4)	0.41969 (14)	0.0535 (11)	
H12A	0.3130	0.1726	0.4396	0.064*	
C13	0.4519 (5)	0.2939 (3)	0.41227 (13)	0.0450 (10)	
C14	0.5843 (5)	0.3161 (3)	0.37735 (13)	0.0463 (10)	
C15	0.6873 (5)	0.4283 (4)	0.39076 (15)	0.0553 (11)	
H15A	0.7681	0.4011	0.4119	0.066*	
H15B	0.7386	0.4587	0.3631	0.066*	
C16	0.5968 (5)	0.5356 (3)	0.41356 (14)	0.0576 (11)	
H16A	0.6712	0.5968	0.4240	0.069*	
H16B	0.5302	0.5737	0.3905	0.069*	
C17	0.4960 (5)	0.4953 (4)	0.45462 (14)	0.0521 (10)	
C18	0.3760 (5)	0.4008 (3)	0.43689 (13)	0.0487 (10)	
H18A	0.3251	0.3666	0.4644	0.058*	
C19	0.2459 (5)	0.4588 (4)	0.40792 (14)	0.0541 (11)	
H19A	0.2902	0.4872	0.3789	0.065*	
H19B	0.1690	0.3963	0.4007	0.065*	
C20	0.1633 (5)	0.5660 (4)	0.43147 (15)	0.0554 (11)	
C21	0.2844 (6)	0.6604 (4)	0.44773 (17)	0.0653 (13)	
H21A	0.3316	0.6990	0.4209	0.078*	
H21B	0.2319	0.7237	0.4655	0.078*	
C22	0.4116 (6)	0.6038 (4)	0.47708 (16)	0.0644 (12)	
H22A	0.3659	0.5766	0.5060	0.077*	
H22B	0.4882	0.6666	0.4843	0.077*	
C23	1.0152 (6)	−0.1466 (5)	0.29607 (17)	0.0815 (16)	
H23A	0.9998	−0.1769	0.3269	0.122*	
H23B	1.0868	−0.0788	0.2967	0.122*	
H23C	1.0573	−0.2107	0.2770	0.122*	
C24	0.8894 (7)	−0.0563 (4)	0.22619 (13)	0.0768 (15)	
H24A	0.7932	−0.0282	0.2128	0.115*	
H24B	0.9316	−0.1216	0.2077	0.115*	
H24C	0.9629	0.0102	0.2273	0.115*	
C25	0.7345 (5)	−0.1141 (4)	0.38010 (14)	0.0589 (12)	
H25A	0.7778	−0.1865	0.3662	0.088*	
H25B	0.6572	−0.1373	0.4026	0.088*	
H25C	0.8162	−0.0685	0.3950	0.088*	
C26	0.7706 (5)	0.1700 (4)	0.42047 (13)	0.0566 (11)	
H26A	0.8343	0.0980	0.4175	0.085*	
H26B	0.6932	0.1570	0.4440	0.085*	
H26C	0.8351	0.2386	0.4288	0.085*	
C27	0.5036 (6)	0.3478 (4)	0.33047 (13)	0.0582 (12)	
H27A	0.4405	0.4199	0.3342	0.087*	
H27B	0.4387	0.2804	0.3210	0.087*	
H27C	0.5820	0.3625	0.3073	0.087*	
C28	0.5997 (6)	0.4369 (4)	0.49226 (14)	0.0668 (12)	
H28A	0.5356	0.4117	0.5178	0.100*	
H28B	0.6753	0.4958	0.5027	0.100*	
H28C	0.6528	0.3668	0.4797	0.100*	
C29	0.0481 (6)	0.6278 (5)	0.39767 (16)	0.0791 (15)	
H29A	−0.0275	0.5687	0.3873	0.119*	
H29B	0.1045	0.6592	0.3715	0.119*	
H29C	−0.0046	0.6940	0.4131	0.119*	
C30	0.0645 (5)	0.5169 (4)	0.47178 (15)	0.0557 (11)	

### Atomic displacement parameters (Å^2^)

**Table:**

	*U*^11^	*U*^22^	*U*^33^	*U*^12^	*U*^13^	*U*^23^
O1	0.123 (3)	0.0579 (19)	0.090 (2)	0.001 (3)	0.019 (3)	−0.0219 (19)
O2	0.081 (2)	0.0426 (15)	0.0673 (18)	−0.0104 (17)	0.0202 (19)	0.0001 (14)
O3	0.084 (2)	0.0523 (18)	0.084 (2)	−0.007 (2)	0.012 (2)	−0.0047 (16)
O4	0.109 (3)	0.0517 (17)	0.080 (2)	0.006 (2)	0.023 (2)	−0.0056 (17)
N1	0.102 (3)	0.053 (2)	0.075 (2)	0.001 (3)	0.008 (3)	−0.011 (2)
N2	0.107 (4)	0.061 (3)	0.079 (3)	0.012 (3)	0.020 (3)	−0.013 (2)
C1	0.069 (3)	0.048 (2)	0.061 (2)	0.002 (3)	0.000 (3)	−0.003 (2)
C2	0.082 (3)	0.044 (2)	0.050 (2)	0.001 (3)	−0.003 (3)	−0.003 (2)
C3	0.086 (4)	0.053 (3)	0.047 (2)	0.009 (3)	0.005 (3)	−0.002 (2)
C4	0.066 (3)	0.058 (3)	0.052 (2)	0.010 (3)	0.006 (2)	−0.002 (2)
C5	0.059 (3)	0.053 (2)	0.048 (2)	0.006 (2)	0.004 (2)	0.0004 (19)
C6	0.054 (3)	0.062 (3)	0.059 (2)	−0.004 (3)	0.009 (2)	0.002 (2)
C7	0.058 (3)	0.054 (2)	0.060 (3)	−0.007 (2)	0.012 (2)	0.000 (2)
C8	0.048 (2)	0.043 (2)	0.047 (2)	−0.003 (2)	0.003 (2)	0.0021 (18)
C9	0.052 (2)	0.041 (2)	0.048 (2)	−0.003 (2)	0.000 (2)	0.0028 (17)
C10	0.053 (2)	0.042 (2)	0.049 (2)	0.006 (2)	0.003 (2)	0.0019 (19)
C11	0.057 (3)	0.044 (2)	0.056 (2)	−0.005 (2)	0.006 (2)	−0.0002 (19)
C12	0.059 (3)	0.045 (2)	0.057 (2)	−0.008 (2)	0.013 (2)	0.000 (2)
C13	0.045 (2)	0.045 (2)	0.045 (2)	−0.002 (2)	0.000 (2)	0.0007 (18)
C14	0.051 (2)	0.040 (2)	0.048 (2)	−0.003 (2)	−0.003 (2)	−0.0002 (18)
C15	0.055 (3)	0.049 (2)	0.062 (3)	−0.013 (2)	0.002 (2)	0.002 (2)
C16	0.060 (3)	0.043 (2)	0.070 (3)	−0.009 (2)	−0.004 (3)	−0.001 (2)
C17	0.050 (2)	0.048 (2)	0.058 (2)	−0.007 (2)	−0.001 (2)	−0.007 (2)
C18	0.051 (2)	0.043 (2)	0.053 (2)	0.002 (2)	−0.003 (2)	−0.0029 (18)
C19	0.054 (3)	0.051 (2)	0.058 (2)	0.002 (2)	−0.004 (2)	0.001 (2)
C20	0.054 (3)	0.048 (2)	0.064 (3)	0.007 (2)	−0.004 (2)	0.002 (2)
C21	0.065 (3)	0.047 (2)	0.084 (3)	−0.001 (2)	0.003 (3)	−0.003 (2)
C22	0.061 (3)	0.050 (2)	0.082 (3)	−0.002 (3)	−0.007 (3)	−0.016 (2)
C23	0.079 (4)	0.083 (4)	0.082 (3)	0.025 (3)	0.007 (3)	−0.004 (3)
C24	0.098 (4)	0.080 (3)	0.052 (2)	0.002 (4)	0.017 (3)	−0.008 (2)
C25	0.075 (3)	0.052 (2)	0.050 (2)	0.008 (3)	0.000 (2)	0.005 (2)
C26	0.061 (3)	0.058 (3)	0.051 (2)	0.005 (2)	−0.005 (2)	0.003 (2)
C27	0.071 (3)	0.053 (2)	0.051 (2)	0.001 (3)	−0.003 (2)	0.0071 (19)
C28	0.066 (3)	0.075 (3)	0.059 (3)	0.005 (3)	−0.009 (3)	−0.009 (2)
C29	0.078 (4)	0.078 (3)	0.082 (3)	0.016 (3)	−0.001 (3)	0.024 (3)
C30	0.053 (3)	0.048 (2)	0.066 (3)	0.008 (2)	−0.012 (2)	−0.001 (2)

### Geometric parameters (Å, °)

**Table:**

O1—N2	1.393 (5)	C15—H15B	0.9700
O1—N1	1.408 (5)	C16—C17	1.531 (5)
O2—C11	1.237 (4)	C16—H16A	0.9700
O3—C30	1.197 (5)	C16—H16B	0.9700
O4—C30	1.336 (5)	C17—C22	1.530 (5)
O4—H4A	0.8200	C17—C18	1.541 (5)
N1—C2	1.304 (5)	C17—C28	1.541 (5)
N2—C3	1.301 (5)	C18—C19	1.528 (5)
C1—C2	1.495 (6)	C18—H18A	0.9800
C1—C10	1.554 (6)	C19—C20	1.526 (5)
C1—H1A	0.9700	C19—H19A	0.9700
C1—H1B	0.9700	C19—H19B	0.9700
C2—C3	1.401 (7)	C20—C21	1.534 (6)
C3—C4	1.501 (6)	C20—C30	1.535 (6)
C4—C23	1.535 (6)	C20—C29	1.542 (6)
C4—C24	1.556 (5)	C21—C22	1.511 (6)
C4—C5	1.578 (6)	C21—H21A	0.9700
C5—C6	1.534 (6)	C21—H21B	0.9700
C5—C10	1.564 (6)	C22—H22A	0.9700
C5—H5A	0.9800	C22—H22B	0.9700
C6—C7	1.527 (6)	C23—H23A	0.9600
C6—H6A	0.9700	C23—H23B	0.9600
C6—H6B	0.9700	C23—H23C	0.9600
C7—C8	1.541 (5)	C24—H24A	0.9600
C7—H7A	0.9700	C24—H24B	0.9600
C7—H7B	0.9700	C24—H24C	0.9600
C8—C9	1.556 (5)	C25—H25A	0.9600
C8—C26	1.554 (5)	C25—H25B	0.9600
C8—C14	1.578 (5)	C25—H25C	0.9600
C9—C11	1.519 (5)	C26—H26A	0.9600
C9—C10	1.565 (5)	C26—H26B	0.9600
C9—H9A	0.9800	C26—H26C	0.9600
C10—C25	1.543 (5)	C27—H27A	0.9600
C11—C12	1.456 (5)	C27—H27B	0.9600
C12—C13	1.330 (5)	C27—H27C	0.9600
C12—H12A	0.9300	C28—H28A	0.9600
C13—C18	1.512 (5)	C28—H28B	0.9600
C13—C14	1.536 (5)	C28—H28C	0.9600
C14—C15	1.556 (5)	C29—H29A	0.9600
C14—C27	1.559 (5)	C29—H29B	0.9600
C15—C16	1.549 (5)	C29—H29C	0.9600
C15—H15A	0.9700		
			
N2—O1—N1	110.5 (3)	C15—C16—H16B	108.9
C30—O4—H4A	109.5	H16A—C16—H16B	107.8
C2—N1—O1	103.6 (4)	C22—C17—C16	111.8 (3)
C3—N2—O1	105.4 (4)	C22—C17—C18	110.1 (3)
C2—C1—C10	109.4 (4)	C16—C17—C18	108.0 (3)
C2—C1—H1A	109.8	C22—C17—C28	106.9 (3)
C10—C1—H1A	109.8	C16—C17—C28	110.0 (4)
C2—C1—H1B	109.8	C18—C17—C28	110.0 (3)
C10—C1—H1B	109.8	C13—C18—C19	111.9 (3)
H1A—C1—H1B	108.2	C13—C18—C17	112.7 (3)
N1—C2—C3	111.2 (4)	C19—C18—C17	113.0 (3)
N1—C2—C1	125.3 (5)	C13—C18—H18A	106.2
C3—C2—C1	123.5 (4)	C19—C18—H18A	106.2
N2—C3—C2	109.2 (5)	C17—C18—H18A	106.2
N2—C3—C4	124.9 (5)	C20—C19—C18	114.2 (3)
C2—C3—C4	125.8 (4)	C20—C19—H19A	108.7
C3—C4—C23	110.4 (4)	C18—C19—H19A	108.7
C3—C4—C24	108.2 (4)	C20—C19—H19B	108.7
C23—C4—C24	107.3 (4)	C18—C19—H19B	108.7
C3—C4—C5	108.3 (4)	H19A—C19—H19B	107.6
C23—C4—C5	115.2 (4)	C19—C20—C21	109.8 (4)
C24—C4—C5	107.2 (3)	C19—C20—C30	109.0 (3)
C6—C5—C10	110.5 (3)	C21—C20—C30	111.9 (4)
C6—C5—C4	111.2 (4)	C19—C20—C29	110.4 (4)
C10—C5—C4	117.1 (3)	C21—C20—C29	109.4 (4)
C6—C5—H5A	105.7	C30—C20—C29	106.3 (4)
C10—C5—H5A	105.7	C22—C21—C20	112.6 (3)
C4—C5—H5A	105.7	C22—C21—H21A	109.1
C7—C6—C5	110.8 (4)	C20—C21—H21A	109.1
C7—C6—H6A	109.5	C22—C21—H21B	109.1
C5—C6—H6A	109.5	C20—C21—H21B	109.1
C7—C6—H6B	109.5	H21A—C21—H21B	107.8
C5—C6—H6B	109.5	C21—C22—C17	114.6 (4)
H6A—C6—H6B	108.1	C21—C22—H22A	108.6
C6—C7—C8	113.8 (3)	C17—C22—H22A	108.6
C6—C7—H7A	108.8	C21—C22—H22B	108.6
C8—C7—H7A	108.8	C17—C22—H22B	108.6
C6—C7—H7B	108.8	H22A—C22—H22B	107.6
C8—C7—H7B	108.8	C4—C23—H23A	109.5
H7A—C7—H7B	107.7	C4—C23—H23B	109.5
C7—C8—C9	110.4 (3)	H23A—C23—H23B	109.5
C7—C8—C26	107.4 (3)	C4—C23—H23C	109.5
C9—C8—C26	109.8 (3)	H23A—C23—H23C	109.5
C7—C8—C14	110.3 (3)	H23B—C23—H23C	109.5
C9—C8—C14	108.5 (3)	C4—C24—H24A	109.5
C26—C8—C14	110.5 (3)	C4—C24—H24B	109.5
C11—C9—C8	107.9 (3)	H24A—C24—H24B	109.5
C11—C9—C10	115.1 (3)	C4—C24—H24C	109.5
C8—C9—C10	118.8 (3)	H24A—C24—H24C	109.5
C11—C9—H9A	104.5	H24B—C24—H24C	109.5
C8—C9—H9A	104.5	C10—C25—H25A	109.5
C10—C9—H9A	104.5	C10—C25—H25B	109.5
C25—C10—C1	108.3 (3)	H25A—C25—H25B	109.5
C25—C10—C9	115.1 (3)	C10—C25—H25C	109.5
C1—C10—C9	106.5 (3)	H25A—C25—H25C	109.5
C25—C10—C5	112.3 (4)	H25B—C25—H25C	109.5
C1—C10—C5	107.3 (3)	C8—C26—H26A	109.5
C9—C10—C5	106.9 (3)	C8—C26—H26B	109.5
O2—C11—C12	119.7 (4)	H26A—C26—H26B	109.5
O2—C11—C9	123.2 (4)	C8—C26—H26C	109.5
C12—C11—C9	117.0 (3)	H26A—C26—H26C	109.5
C13—C12—C11	124.7 (4)	H26B—C26—H26C	109.5
C13—C12—H12A	117.7	C14—C27—H27A	109.5
C11—C12—H12A	117.7	C14—C27—H27B	109.5
C12—C13—C18	119.1 (4)	H27A—C27—H27B	109.5
C12—C13—C14	120.5 (4)	C14—C27—H27C	109.5
C18—C13—C14	120.3 (3)	H27A—C27—H27C	109.5
C13—C14—C15	112.1 (3)	H27B—C27—H27C	109.5
C13—C14—C27	106.2 (3)	C17—C28—H28A	109.5
C15—C14—C27	107.0 (3)	C17—C28—H28B	109.5
C13—C14—C8	109.3 (3)	H28A—C28—H28B	109.5
C15—C14—C8	110.3 (3)	C17—C28—H28C	109.5
C27—C14—C8	111.9 (3)	H28A—C28—H28C	109.5
C16—C15—C14	114.7 (3)	H28B—C28—H28C	109.5
C16—C15—H15A	108.6	C20—C29—H29A	109.5
C14—C15—H15A	108.6	C20—C29—H29B	109.5
C16—C15—H15B	108.6	H29A—C29—H29B	109.5
C14—C15—H15B	108.6	C20—C29—H29C	109.5
H15A—C15—H15B	107.6	H29A—C29—H29C	109.5
C17—C16—C15	113.2 (3)	H29B—C29—H29C	109.5
C17—C16—H16A	108.9	O3—C30—O4	121.7 (4)
C15—C16—H16A	108.9	O3—C30—C20	125.5 (4)
C17—C16—H16B	108.9	O4—C30—C20	112.8 (4)

### Hydrogen-bond geometry (Å, °)

**Table:**

*D*—H···*A*	*D*—H	H···*A*	*D*···*A*	*D*—H···*A*
O4—H4A···O2^i^	0.82	2.04	2.787 (4)	151
C1—H1A···O2	0.97	2.36	2.969 (5)	120
C25—H25B···O2	0.96	2.39	3.021 (5)	123

Symmetry codes: (i) *x*−1/2, −*y*+1/2, −*z*+1.

## References

[bb1] Bruker (1997). *XSCANS* Bruker AXS Inc., Madison, Wisconsin, USA.

[bb2] Finney, R. S. H. & Tarknoy, A. L. (1960). *J. Pharm. Pharmacol.* **12**, 49–58. 10.1111/j.2042-7158.1960.tb12629.x 13822948

[bb3] Sheldrick, G. M. (2008). *Acta Cryst.* A**64**, 112–122. 10.1107/S0108767307043930 18156677

[bb4] Su, X. D., Lawrence, H., Ganeshapillai, D., Cruttenden, A., Purohit, A., Reed, M. J., Vickera, N. & Potter, B. V. L. (2004). *Bioorg. Med. Chem.* **12**, 4439–4457. 10.1016/j.bmc.2004.06.008 15265495

[bb5] Yoshida, K., Furihata, K., Habe, H., Yamane, H. & Omori, T. (2001). *Biotechnol. Lett.* **23**, 1619–1624.

[bb6] Yu, D., Sakurai, Y., Chen, C. H., Chang, F. R., Huang, L., Kashiwada, Y. & Lee, K. H. (2006). *J. Med. Chem.* **49**, 5462–5469. 10.1021/jm0601912 PMC2512972 16942019

